# Necessity of Swallow Evaluations in the Elderly Prior to Capsule Endoscopy

**DOI:** 10.7759/cureus.24138

**Published:** 2022-04-14

**Authors:** Polina Gaisinskaya, Kai Yoshinaga, Oscar L Hernandez

**Affiliations:** 1 Internal Medicine, Florida Atlantic University, Boca Raton, USA

**Keywords:** geriatrics and internal medicine, capsule complication, failed colonoscopy, esophagogastroduodenoscopy (egd), airway foreign body, small bowel capsule endoscopy, capsule endoscopy

## Abstract

Capsule endoscopy (CE) is a convenient and minimally invasive form of gastrointestinal visualization that has found increased favor as technology has allowed for greater quality imaging. This case addresses some of the issues with this modality emphasizing the need to assess for the contraindication of dysphagia. In this case, we describe a frail patient who aspirated a capsule endoscopy while being evaluated for positive fecal occult blood with concomitant anemia after admission for a stroke alert.

## Introduction

Capsule endoscopy (CE) is a frequent imaging modality used by gastroenterologists when conventional endoscopy is not possible or inconclusive to visualize all necessary portions of the gastrointestinal tract. This modality is beneficial and practical in the workup of many structural gastrointestinal issues, from ulcers to masses. Some possible complications of CE include capsule retention or aspiration, but they occur in less than 2% of examinations [1]. In general, aspiration occurs with misdirection of gastric or oropharyngeal contents into the airway. Aspiration can be precipitated by many risk factors including dysphagia, respiratory distress, or altered mental status. Advancements in medical care have led to an increased number of geriatric individuals who are disproportionately affected by some of these risk factors. In these individuals, aspiration and dysphagia incidence is expected to significantly impact both healthcare cost as well as the quality of life [2]. 

Although most cases cause mild respiratory distress, this complication can be life threatening and, with extra precautionary steps, can be avoided. Swallow evaluations are a common method of evaluating frail and elderly patients in a safe and controlled manner, and observer interrater reliability of such a study is adequate for assessing aspiration risk. Swallow studies can reasonably be conducted for patients before capsule endoscopy, as their cost and availability are not excessive. We present an elderly gentleman who aspirated a capsule endoscopy into his bronchi without significant symptoms.

This case was previously presented as a poster at the American College of Gastroenterology Annual Meeting, Las Vegas, United States, in October 2021 [3].

## Case presentation

A 92-year-old male with a past medical history of gout, myelodysplastic syndrome, chronic kidney disease, and iron deficiency anemia presented to the hospital with blurred vision and slurred speech over the previous two days. The patient was initially admitted for a transient ischemic attack workup, which was unremarkable. Medication history was significant for iron supplementation. Gastroenterology was consulted for evaluation of positive fecal occult blood tests and acute on chronic anemia. Of note, on outpatient routine blood work his hemoglobin was 9.3 g/dL the week prior and was found to be 7.9 g/dL at presentation. Esophagogastroduodenoscopy (EGD) and colonoscopy were unrevealing, so the patient underwent a CE. During the administration of the capsule, the patient felt as if he wasn’t able to swallow the capsule easily but felt it went down after drinking water. He remained without any respiratory symptoms and maintained an oxygen saturation above 94% in room air. The live viewer demonstrated the capsule in the airway the next day, which initially was not confirmed after placement via the live viewer. A subsequent chest x-ray confirmed the capsule in the airway. The patient underwent a bronchoscopy for successful retrieval. Due to risks of recurrent procedures, the patient's power of attorney decided to no longer explore the cause of the patient's anemia. The patient continued under conservative management and was discharged following hemoglobin stabilization without any further signs of bleeding.

**Figure 1:**
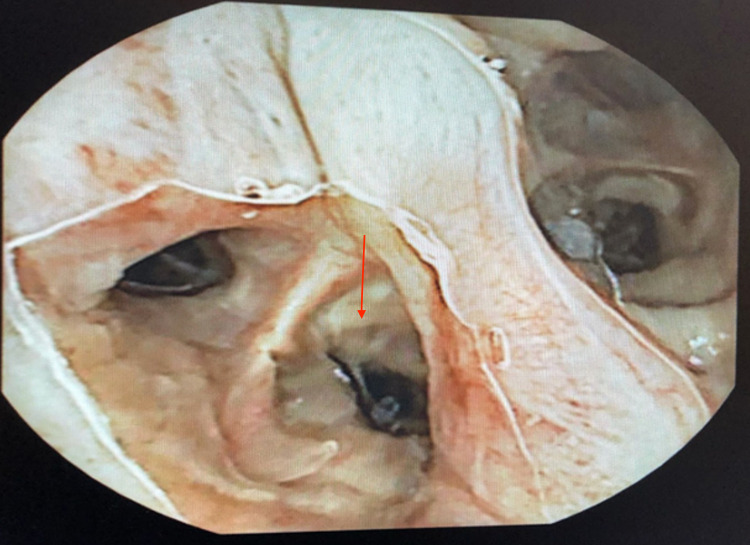
Capsule endoscopy live viewer demonstrated the capsule in the airway, bronchus shown (arrow)

**Figure 2:**
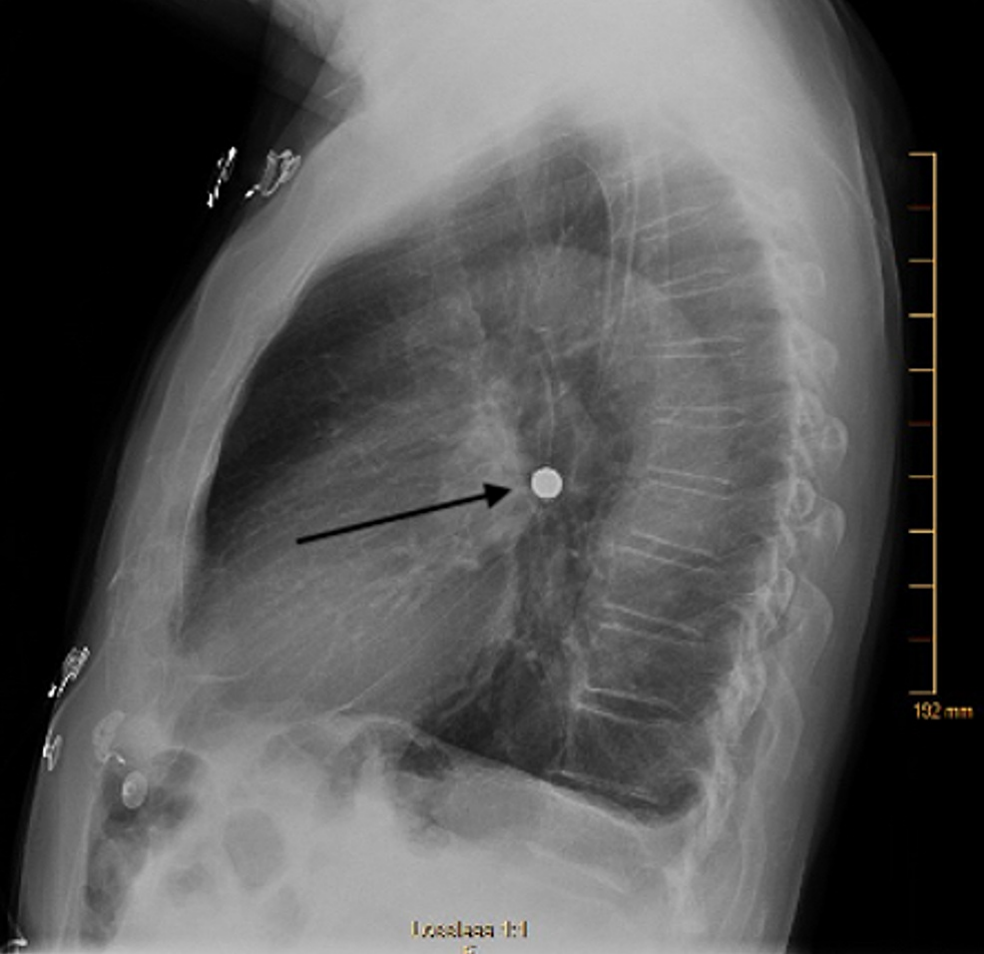
Lateral view of chest x-ray, which was obtained after live viewer demonstrated bronchus

## Discussion

Swallowing difficulty is one of the contraindications for capsule endoscopy. Upon reviewing manufacturing websites of the capsules, such as Medtronic (Medtronic plc, Dublin, Ireland), it was found that they raise caution to providers to be aware of patients with known or potential swallowing disorders. In patients without a known history of dysphagia but deemed at high risk for aspiration events such as those with a history of cerebrovascular events and advanced age, swallow evaluation prior to CE, if feasible, may decrease the risk of aspiration. With aging, the skeletal muscle, masticatory, as well as lingual muscle decrease in strength [4]. This alone is a significant risk factor for aspiration. Some healthier elders (those above 65 years of age) may have functional senescent swallowing until a certain trigger, such as acute illness or medication adverse effects, causes disruption and results in dysphagia. Second-line gastroscopy with direct placement is an alternative option for those patients who have a significant risk for aspiration. Screening for aspiration on admission can aid in pre-endoscopic planning. Gastroenterologists are able to prepare a CE to be ready when the patients undergo conventional upper endoscopy and colonoscopy, in the case that the source of pathology is not found. The CE can then be placed safely during the time of the initial procedures, decreasing wasted time, resources, adverse events, and costs. Some studies have been conducted to estimate the risk of aspiration. One study looked at a series of 733 CE procedures, where 11 patients had difficulty or were unable to swallow the capsule. One patient aspirated the capsule, followed by spontaneous expulsion by coughing [5].

The gold standard for diagnosis of aspiration and dysphagia is videofluoroscopy (VFS), as endoscopic evaluation is limited to assessing motility. VFS or modified barium swallow study is a radiographic procedure that provides a direct, dynamic view of oral, pharyngeal, and upper esophageal function [5]. In the acute setting, this may further delay evaluation via CE. Smith et al, in a double-blind observational study, used the combination of bedside swallow and monitoring of oxygen saturation compared to VFS in patients with acute stroke. They found that 86% of their patients were detected for dysphagia via saturation assessments in combination with bedside swallow, which was statistically significant [2]. A combination of bedside swallow and simultaneous oxygen saturation monitoring showed an increase in the positive predictive value for the diagnosis of aspiration. This may be a cost-effective and time-saving alternative that will help mitigate the risk of CE aspiration in the elderly and prevent delay of gastrointestinal exploration. If there is uncertainty regarding swallowing ability then a postpyloric placement of the capsule, ideally during the time of the first procedure, should be considered. In these cases, the placement of the capsule can be done via a polypectomy snare, oroesophageal overtube, or foreign object retrieval devices [6,7]. In cases of gastric outlet obstruction, the overtube is preferred to place the capsule directly into the duodenum and has been found to be safe and effective. The downside to this approach is that it likely requires systemic sedation [8]. 

## Conclusions

Although patients may compensate for the natural course of aging when it comes to swallowing function, vigilance should remain high when subjecting these individuals to CE. When additional risk factors are present, such as a possible cerebrovascular event, precautions should be taken prior to initiating this study. Proper screening of individuals prior to CE can aid in peri-endoscopic planning, increase cost effectiveness, and decrease adverse events. Further research is needed on the sensitivity and specificity of the combination oxygen saturation and bedside swallow when compared to VFS prior to CE in high risk, elderly patients. 
